# Geographical Discrimination of Xinhui Citri Reticulatae Pericarpium by DART-QTOF-MS

**DOI:** 10.3390/foods15081361

**Published:** 2026-04-14

**Authors:** Ximei Wu, Qunjie Feng, Wenbo Duan, Wei Tong, Jian Wen, Gangqiang Ding

**Affiliations:** 1 NHC Specialty Laboratory of Food Safety Risk Assessment and Standard Development, Guangdong Provincial Center for Disease Control and Prevention, Guangzhou 511430, China; 2 Carbon Neutrality and Climate Change Thrust, Society Hub, The Hong Kong University of Science and Technology (Guangzhou), Guangzhou 511453, China

**Keywords:** Citri Reticulatae Pericarpium, DART-QTOF-MS, geographical origin discrimination, 2-indolinone, rapid fingerprinting

## Abstract

Xinhui Citri Reticulatae Pericarpium (CRP, “Chenpi”) is highly valued but is frequently challenged by origin-related adulteration and mislabeling. In this study, a rapid fingerprinting strategy based on direct analysis in real-time quadrupole time-of-flight mass spectrometry (DART-QTOF-MS) coupled with chemometric analysis was developed for the geographical characterization of CRP. DART-QTOF-MS enabled fast acquisition of mass spectral fingerprints with minimal sample preparation, and characteristic compounds were tentatively assigned on the basis of accurate mass and library matching. Comparative analysis showed that the high-mass region was dominated by polymethoxylated flavones and exhibited relatively limited between-region variation. In contrast, the low-mass region contained more evident origin-related differences and provided more informative variables for classification. Among the low-molecular-weight compounds, methyl N-methylanthranilate was markedly enriched in Xinhui samples, and 2-indolinone was identified as a promising candidate marker and further confirmed in CRP extracts by UPLC–MS/MS. OPLS-DA based on selected low-molecular-weight markers supported the discrimination of core Xinhui CRP and non-core Xinhui CRP. Overall, these results demonstrate the potential of DART-QTOF-MS as a screening tool for CRP origin authentication and highlight the value of low-molecular-weight markers for future quality-control applications.

## 1. Introduction

Citri Reticulatae Pericarpium (CRP, “Chenpi”) is the dried, aged pericarp of the tangerine (Citrus reticulata Blanco and its cultivated varieties) in the family Rutaceae [1]. In traditional Chinese medicine and food culture, CRP is categorized into general “Chenpi” and the high-quality “Guang Chenpi” produced in Guangdong [2,3]. Notably, the Guang Chenpi from Xinhui (Jiangmen, Guangdong) is regarded as the most authentic and premium grade [4,5]. It is celebrated for its special aroma and efficacy, and is one of the famed “Three Treasures of Guangdong” (alongside aged ginger and rice straw). In traditional Chinese medicine, Chenpi is a Qi-regulating herb used to strengthen the spleen and alleviate digestive and respiratory ailments; it has been used for centuries for conditions such as nausea, indigestion, cough, and excessive phlegm [1]. However, the current market for Xinhui Chenpi is highly heterogeneous, and fraudulent practices such as counterfeiting, substitution with inferior peels, and mislabeling of origin occur frequently [6]. Because genuine Xinhui Chenpi commands a premium price, fraudulent practices have become common: lower-quality peels from other regions are often misrepresented as Xinhui products to boost profits [6]. Such adulteration not only misleads consumers but also threatens the reputation and sustainable development of the Xinhui Chenpi industry. To protect consumers and uphold the quality of this traditional product, it is necessary to investigate the characteristic chemical profile of Chenpi from different regions and establish robust methods for origin authentication [7].

Recent phytochemical investigations have shown that CRP is rich in flavonoids, along with an array of other secondary metabolites [4,8,9,10]. Over sixty flavonoid compounds (including flavanone glycosides and polymethoxylated flavones (PMFs)) have been isolated and identified from Chenpi [1]. Among these, hesperidin, a dominant flavanone O-glycoside, has been adopted as the principal chemical marker for CRP in the Chinese Pharmacopoeia (2015 edition) [11]. Prior research indicates that Xinhui chenpi can differ from chenpi of other origins in the levels of certain flavonoids and polymethoxyflavones, which may influence its biological activities [12]. For example, Luo et al. observed significantly higher polymethoxyflavone content in chenpi from the *C.* *reticulata* “Chachi” cultivar (originating in Xinhui) compared to other cultivars [13]. These findings underscore that the flavonoid fingerprint of Chenpi can serve as a distinguishing feature of its botanical origin and variety. Nonetheless, conventional chromatographic methods such as HPLC and GC-MS remain the main approaches for Chenpi characterization, but their time-consuming pretreatment and separation steps hinder high-throughput authenticity screening [14,15,16,17,18,19]. To improve geographical authentication of Chenpi, recent studies have increasingly adopted data-driven strategies. For example, UHPLC-QTOF-MS-based metabolomics combined with chemometrics has been used to classify Chenpi across cultivars and producing regions [20,21], and DNA barcoding has also been reported to distinguish authentic Guang Chenpi with high accuracy [22]. Nevertheless, these approaches still face practical constraints: chromatographic fingerprints can be influenced by biological and processing variability and may lack universally robust markers, while DNA-based identification typically requires specialized expertise and high-quality genetic material, which may not always be preserved in aged peel products. Therefore, there remains an obvious need for a rapid, reliable, and broadly applicable method to identify the geographical origin of Chenpi in a high-throughput manner.

Direct Analysis in Real-Time mass spectrometry (DART-MS) is an ambient ionization technique that offers a rapid alternative for analyzing complex samples with minimal preparation [23]. In DART-MS, analytes are ionized in an open environment at atmospheric pressure by a stream of excited-state gas, and the desorbed ions are then introduced directly into the mass spectrometer inlet [23]. This approach eliminates the need for dissolving the sample or performing chromatographic separation, allowing solid or liquid samples to be analyzed as is or after only minimal pretreatment. Compared with traditional GC-MS, which often produces extensive fragmentation, DART-MS yields prominent molecular ions (e.g., [M + H]^+^) with little in-source fragmentation [24]. The spectra are thus simpler to interpret, as intact parent masses are observed rather than complex fragment patterns [24]. When coupled to a high-resolution QTOF-MS, DART can rapidly generate prominent molecular ions, while the TOF detector provides accurate mass measurements with high resolving power [23]. Overall, DART coupled with QTOF-MS enables rapid acquisition of information-rich chemical fingerprints, making it well-suited for high-throughput authenticity assessment of herbal material [25,26].

In this study, we employ DART-QTOF-MS to analyze CRP samples from Xinhui and other regions with the following aims: (i) to establish a fast, efficient DART-MS fingerprinting method for Chenpi; (ii) to compare the chemical profiles of Chenpi from different geographical origins; and (iii) to discover marker compounds capable of distinguishing authentic Xinhui Chenpi from non-Xinhui products. By developing a rapid chemical authentication strategy, we hope to provide technical support for Chenpi origin verification and contribute to the improvement of its quality standard system.

## 2. Materials and Methods

### 2.1. Sample Collection

A total of 93 batches of CRP samples were collected from major production areas in Guangdong, Hunan, Guangxi, Anhui, and Zhejiang provinces of China. All samples were derived from the same harvest year and were aged for 2 years to minimize vintage-related variation. Among the collected samples, 67 batches were from Xinhui and were used for the model of subregional discrimination within Xinhui, including 29 batches from the recognized core production regions (Meijiang, Dongjia, Tianma, and Chakeng) and 38 batches from the non-core production regions of Xinhui (Siqian, Yamen and Gujing). The remaining CRP samples, collected from non-Xinhui regions of Guangdong and from other provinces, were used for broader comparative analysis. Detailed sample information is provided in Table S1 of the Supplementary Material. All samples were authenticated by experienced CRP practitioners based on appearance, aroma, and labeling information, and then were stored in a cool, dry place until analysis.

### 2.2. Chemicals and Reagents

A reference standard of 2-indolinone (≥99.8% purity) was obtained from the National Institutes for Food and Drug Control (Beijing, China). HPLC-grade methanol was purchased from Sigma-Aldrich (St. Louis, MO, USA). Formic acid (98–100%) was from Merck KGaA (Darmstadt, Germany).

### 2.3. Sample Preparation

A total of 93 independent commercial dried CRP batches were collected for each geographical region. Each batch represented a single lot from a specific production from the same origin. Within each batch, multiple peel pieces were composited, pooled and homogenized to obtain a representative sample. The Xinhui batches were further classified, according to the commonly recognized regional division adopted in previous studies [27], into core production regions (R: Meijiang, Dongjia, Tianma, and Chakeng) and non-core production regions (S: Siqian, Yamen, and Gujing). All samples were derived from the same harvest year.

For each batch (independent CRP lot), multiple peel pieces were pooled to obtain a representative composite sample. Before extraction, the pooled material was cut into small pieces and ground into a homogeneous fine powder, with sieving performed when necessary to improve sample uniformity. Three independent 2.5 g aliquots were accurately weighed from each batch composite, transferred into separate 50 mL centrifuge tubes, and extracted in parallel with 25 mL of 10% (*v*/*v*) aqueous methanol. The mixture was vigorously shaken for 10 min and then subjected to ultrasonic extraction for 15 min. The suspension was centrifuged at 8000 rpm for 10 min. The resulting supernatant was filtered through a membrane filter and used directly for DART-QTOF-MS analysis. The extract was diluted 10-fold and then analyzed by UHPLC-MS/MS.

### 2.4. DART-QTOF-MS Analysis

DART-QTOF-MS analysis was performed using a DART-SVP™ ion source (IonSense, Saugus, MA, USA) coupled to a Waters Xevo G2-XS QTOF mass spectrometer (Waters Corp., Milford, MA, USA). Sample introduction was performed with Dip-it glass-rod samplers (IonSense). A 2 µL aliquot of each CRP methanolic extract was pipetted onto the tip of a Dip-it glass rod and immediately placed into the autosampler for analysis, with each extract analyzed in five replicate measurements to assess instrumental repeatability. The automated sample holder was moved vertically into the stream of ionizing gas at a programmed speed of 0.6 mm/s, allowing multiple samples to be analyzed sequentially in a single run. Helium was used as the DART ionizing gas at a flow rate of 2 L/min, and the DART gas temperature was set to 400 °C. The grid electrode voltage was maintained at 50 V. Dip-it samplers with loaded samples were positioned at a distance of approximately 2.3 cm from the DART exit and in front of the MS sampling orifice.

The QTOF-MS was operated in positive-ion mode with a resolving power of approximately 25,000 FWHM. Leucine-enkephalin was used as a lock-mass reference to ensure mass accuracy within the required tolerance during data acquisition. The capillary (spray) and cone voltages were set to 2.0 and 30 V, respectively, to obtain DART spectra containing both [M + H]^+^ precursor ions and characteristic fragment ions. The acquisition range was 50–600 Da. The desolvation temperature was 500 °C, with a desolvation gas flow of 800 L/h and a cone gas flow of 100 L/h; the source temperature was 250 °C. All data were acquired in full-scan positive-ion mode. Each sample was analyzed in triplicate, using three independently prepared parallel extracts, to ensure reproducibility of the spectral profiles.

### 2.5. UHPLC–MS/MS Analysis of 2-Indolinone

Targeted confirmation of 2-indolinone was performed by UPLC–MS/MS using Shimadzu Nexera LC-40 UHPLC coupled to a Shimadzu 8060 triple-quadrupole mass spectrometer (Shimadzu, Tokyo, Japan) equipped with an electrospray ionization (ESI) source. LabSolutions software (v1.6, Shimadzu) was used for instrument control, data acquisition, and processing. Chromatographic separation was achieved on a BEH C18 column (100 mm × 2.1 mm i.d., 1.7 μm; Waters) maintained at 30 °C, with a flow rate of 0.3 mL/min and an injection volume of 2 μL. The mobile phase consisted of solvent A (water containing 0.05% formic acid) and solvent B (methanol containing 0.05% formic acid), using the following gradient: 0 min, 10% B; 7 min, 70% B; 10.5 min, 99% B; 14 min, 99% B; 15 min, 10% B (total run time 16 min). MS/MS detection was carried out in multiple reaction monitoring (MRM) mode in positive-ion ESI. Two characteristic transitions of 2-indolinone were monitored, with the most intense transition used for quantification and the second transition serving as a qualifier. The ion spray voltage was set to +5500 V, the source temperature was 300 °C, the desolvation line temperature was 250 °C, and the heated nitrogen and desolvation gas flow rates were both 10 L/min.

### 2.6. Data Processing and Chemometric Analysis

DART-QTOF-MS data were processed using MassLynx software (version 4.1; Waters Corporation, Milford, MA, USA). Information on known CRP constituents was collected from the literature and from online databases such as ChemicalBook and ChemSpider. Based on these sources, an in-house compound library was established in UNIFI software (version 1.8) (Waters Corporation, Milford, MA, USA), and the acquired high-resolution MS data were imported into UNIFI for library searching and matching. Tentative identification of characteristic compounds was performed by comparing the accurate mass, isotopic pattern, and fragment ions of detected peaks with the reference library entries. After background subtraction, ion intensities of the selected characteristic *m*/*z* features were extracted from the peak apex and from a defined stable time window around the apex for each DART sampling event. Potential markers with a detection frequency > 80% and an RSD < 30% were retained, and their mean ion intensities were exported to Excel. Signal intensities were normalized to the ion intensity of nobiletin (*m*/*z* 403.1354), which showed the highest response among the detected compounds. Prior to OPLS-DA modeling in SIMCA, the normalized data were Pareto-scaled.

Orthogonal partial least squares–discriminant analysis (OPLS-DA) was performed in SIMCA (v14.1, Umetrics, Umeå, Sweden) to explore similarities and differences among Xinhui CRP samples from different geographical origins. Model robustness was assessed using internal cross-validation and permutation testing, consistent with recommended validation practices for supervised classification in metabolomics to mitigate the risk of overfitting [28]. Model fit and predictive performance were evaluated using the cumulative R^2^Y and Q^2^ values, and model significance was further assessed by permutation testing.

## 3. Results

### 3.1. Profiling of Characteristic Compounds in CRP by DART-QTOF-MS

CRP is a traditional medicinal and edible material with a complex chemical composition, in which flavonoids and volatile constituents are generally regarded as major bioactive components. CRP samples from different geographical origins were analyzed under the optimized DART-QTOF-MS conditions, and a representative TIC of a methanolic extract is shown in Figure S1. Through extraction and alignment of ion features from the acquired high-resolution mass spectra, combined with accurate-mass database matching, isotope pattern evaluation, specialized software queries, and cross-referencing with the literature, 19 characteristic compounds with relatively high signal intensities were tentatively identified [3,29,30,31].

**Figure 1:**
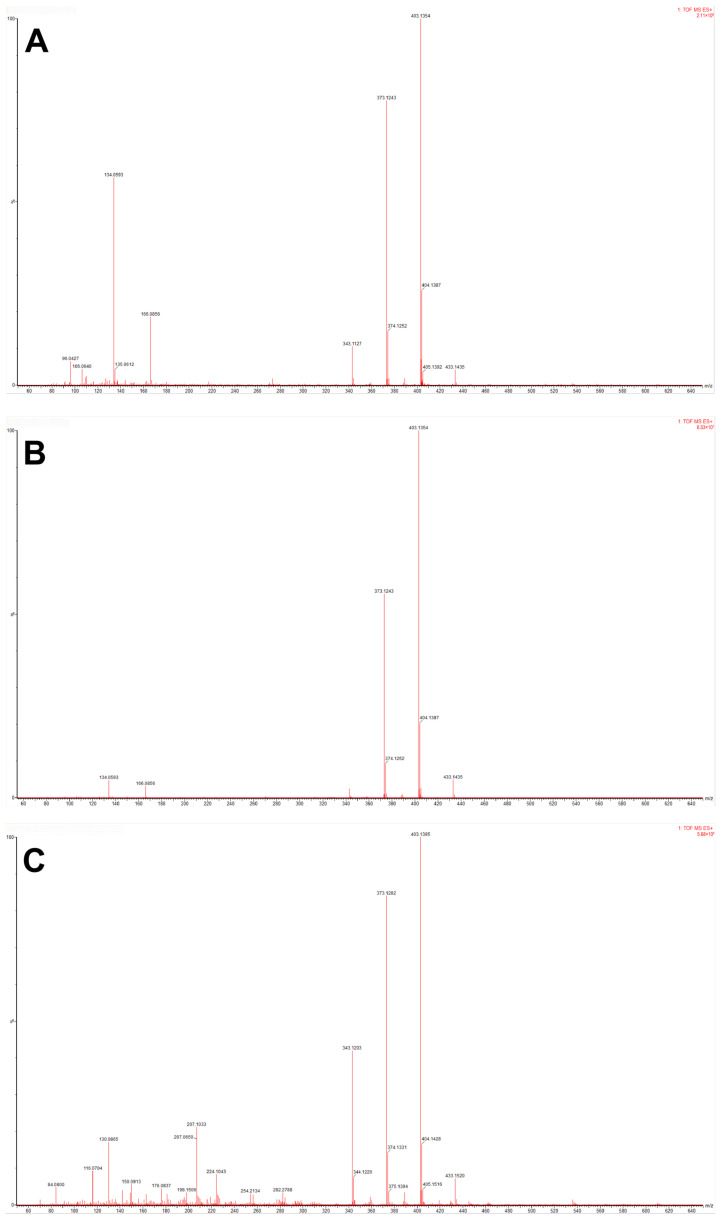
Representative DART-QTOF-MS fingerprint spectra of CRP from different origins: (**A**) Xinhui, Tianma; (**B**) Xinhui, Sanjiang; (**C**) Hunan.

Comparative analysis of the DART-QTOF-MS fingerprints revealed a high degree of compositional consistency across origins. As shown in Figure 1A–C, the mass spectra from different regions displayed highly similar major peaks, and the overall similarity of the chemical fingerprints within the *m*/*z* 300–600 range remained consistently high.

### 3.2. Differential LMW Compounds Among CRP from Different Production Regions

In contrast to the high-mass region, more pronounced regional differences were observed for low-molecular-weight (LMW) ions. Comparison of DART-QTOF-MS fingerprints revealed that, in the *m*/*z* 100–300 range, CRP extracts from Xinhui (Figure 1A,B) exhibited clearly stronger signals than those from other provinces (Figure 1C).

Database searches and the literature review suggested that the more intense LMW signals could be assigned to methyl 2-methylaminobenzoate (*m*/*z* 166.0856), 2-indolinone (*m*/*z* 134.0593), 2-acetylpyrrole (*m*/*z* 110.0576), Octanal (*m*/*z* 130.0865), 5,6-dihydroxyindole (*m*/*z* 150.0888), and related compounds. Representative mass spectra of the potential marker ions are shown in Figures S2–S4, including the full *m*/*z* 50–650 range (Figure S2), the enlarged low-*m*/*z* region (*m*/*z* 50–320; Figure S3), and the high-*m*/*z* region (*m*/*z* 440–650; Figure S4). Among these, 2-indolinone ([M + H]^+^, 134.0593) and methyl N-methylanthranilate ([M + H]^+^, 166.0586) were relatively enriched in Xinhui CRP. In particular, 2-indolinone showed pronounced signals in samples from multiple core production regions of Xinhui, whereas the corresponding peaks in CRP from other provinces were weak or barely detectable (Figure 1), suggesting that it may serve as a potential marker compound for Xinhui Guang Chenpi.

**Figure 2:**
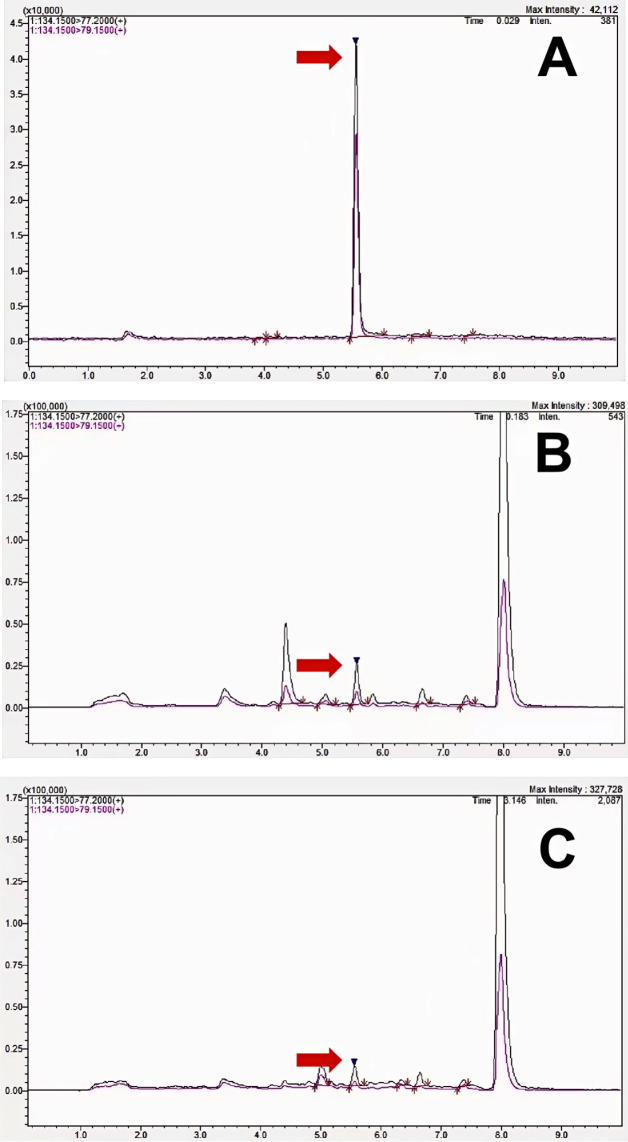
UPLC–MS/MS MRM chromatograms of 2-indolinone in standards and CRP extracts. The black trace and labels represent the quantifier transition (134.1500/77.2000), and the purple trace and labels represent the qualifier transition (134.1500/79.1500). Black inverted triangles indicate the corresponding 2-indolinone peaks in the chromatograms. (**A**) 2-indolinone standard (23 μg/L); (**B**) CRP extract from Sanjiang Town (16.2 μg/L); (**C**) CRP extract from Gujing Town (9.05 μg/L).

To confirm its presence in the CRP matrix, a 2-indolinone reference standard was analyzed by UHPLC–MS/MS together with CRP extracts. Two specific transitions were monitored: the MS/MS (134.1500/77.2000) and MS/MS (134.1500/79.1500). As shown in Figure 2, the retention time and product-ion ratios of the monitored MRM transitions in the CRP extracts matched those of the standard, confirming the identity of 2-indolinone. The contents of 2-indolinone in samples from different origins are shown in Table S2.

### 3.3. Partial Least Squares–Discriminant Analysis of Characteristic Compounds for Discrimination Between Core and Non-Core Production Regions Within Xinhui

Multivariate statistical analysis has been widely used for varietal and geographical discrimination of agricultural products. In CRP research, previous studies have mainly focused on differentiating Guang CRP from products of other provinces, whereas discrimination between core and non-core production regions within Xinhui remains less explored. To improve subregional discrimination, OPLS-DA was first performed using the mass spectrometric signal intensities of eight higher-molecular-weight (HMW) compounds (compounds No. 12–19 in Table S3) identified in the previous analyses. However, this preliminary model did not clearly distinguish samples from the core and non-core production regions, as shown by the OPLS-DA score plot (Figure S5) and prediction plot (Figure S6). A possible explanation is that tangeretin, 3′,4′,5,7-tetramethoxyflavone, alkaloid glycosides, hesperidin, gardenin B, and other similar flavonoids are widely distributed and present at comparable levels across different geographical origins, making them unsuitable as discriminatory markers for Guang Chenpi from different core production areas. Moreover, the high-molecular-weight compounds listed in Table S3 may be fragmentation products of these PMFs, further limiting their contribution to regional discrimination.

**Figure 3:**
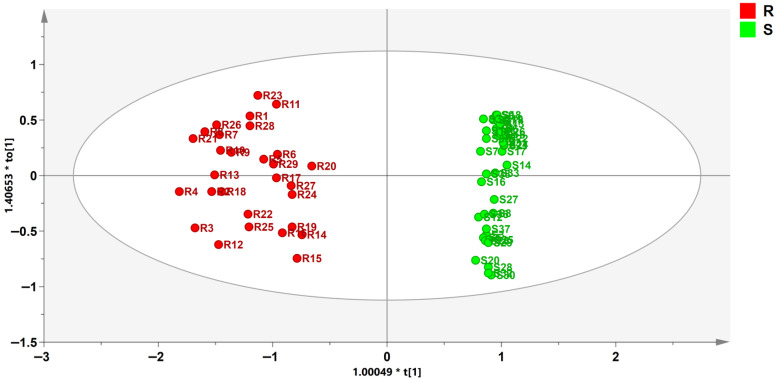
OPLS-DA score plot based on the DART–QTOF–MS signal intensities of 14 potential marker compounds in Citri Reticulatae Pericarpium samples from different Xinhui production regions (R, core production regions: Meijiang, Dongjia, Tianma, and Chakeng; S, non-core production regions: Siqian, Yamen, and Gujing).

**Figure 4:**
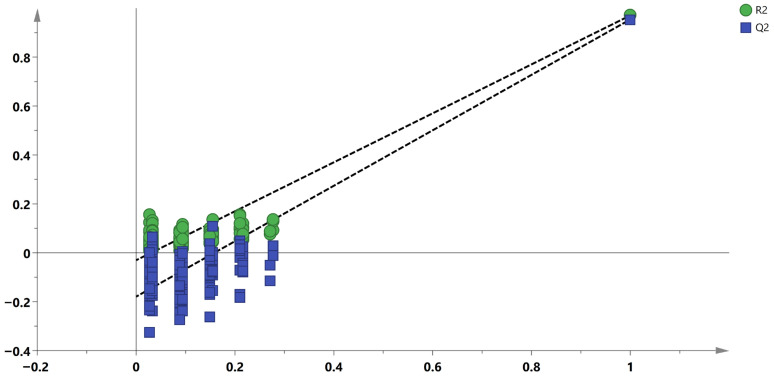
Permutation test of the OPLS-DA model based on 14 potential marker compounds.

Therefore, fourteen low-molecular-weight compounds were selected to reconstruct the OPLS-DA model for screening potential geographical origin markers. The 14 potential marker compounds included in the reconstructed model are summarized in Table 1. The revised model showed more pronounced between-group differences (Figure 3), with cumulative R^2^Y and Q^2^ values of 0.959 and 0.943, respectively. Model validity was further confirmed by a 200-permutation test, which showed no evidence of overfitting (*p* < 0.01) and a Q^2^ intercept of −0.177 (Figure 4). The variable importance in projection (VIP) values of the 14 compounds are shown in Table 1, with 2-indolinone emerging as the most influential variable in the model. In addition, 2-indolinone, methyl N-methylanthranilate, and 2-acetylpyrrole showed relatively higher abundances in samples from the core production region.

## 4. Discussion

This study demonstrates that DART-QTOF-MS, with minimal sample preparation, can rapidly generate information-rich fingerprints for CRP and can contribute to geographical discrimination when combined with OPLS-DA. In the present dataset, ions in the higher-mass window (approximately *m*/*z* 350–600) showed relatively limited between-region variation and only modest discriminatory value, whereas ions in the lower-mass window (approximately *m*/*z* 100–350) tended to show more evident region-dependent differences and provided comparatively more informative variables for classification.

The stability of the HMW region was largely driven by polymethoxylated flavones (PMFs), including tangeretin, tetramethoxyflavone and alkaloid glycosides, which were consistently detected at high relative abundances in all samples. This agrees with prior LC-MS/UHPLC-MS-based phytochemical profiling of CRP and related citrus peels, where PMFs represent dominant and widely distributed secondary metabolites [30,32,33]. Earlier work also proposed that highly methoxylated flavones (≥4 methoxy substituents) are among the key constituents associated with “Guang Chenpi” authenticity [20]. However, the present data indicate that, at least for the sample set analyzed, PMFs form a conserved core chemical signature with only limited region-related variation, so the DART fingerprints in the *m*/*z* 350–600 range showed only a modest tendency toward separation rather than strong origin-specific clustering. This observation is consistent with the concept that citrus flavonoid biosynthesis, particularly PMF formation, is strongly governed by genetic and transcriptional regulation. An integrated multi-omics study on Citrus reticulata cv. Chachiensis mapped the biosynthetic network underlying PMF production and demonstrated that O-methyltransferase activity and upstream transcription factors (e.g., AP2/ERF, bZIP, WRKY) play central roles in controlling PMF accumulation [7]. Large-scale citrus metabolomic surveys likewise support genotype-dominant patterning of major secondary metabolites across diverse cultivars, including species-specific metabolic signatures and limited within-group variation for certain metabolite ratios [34,35]. Taken together, these lines of evidence provide a mechanistic rationale for why HMW PMFs, despite structural diversity, showed only minor quantitative differences among regions in DART-QTOF-MS and therefore contributed only modestly to geographical authentication in the present study.

While genetics likely sets the baseline biosynthetic “blueprint”, environmental conditions can still modulate pathway expression and downstream metabolism, especially for stress-responsive and more labile small molecules. For example, abiotic stress linked to local edaphic and hydrological conditions (e.g., saline influence) has been associated with altered expression of stress-responsive transcriptional regulators and shifts in secondary metabolism in the Xinhui production area [36]. Beyond CRP, metabolomics studies in citrus fruits from different locations have shown that sugars, amino acids, and subsets of flavonoids can vary with both genetic background and environmental drivers such as temperature, light intensity, and soil nutrients [37]. In our dataset, the conserved PMF pattern suggests that environmental variation may imprint more strongly on the LMW chemical space than on highly methylated flavones, making LMW features more informative for tracing origin.

Consistent with this expectation, the low-mass region (*m*/*z* 100–350) exhibited relatively more evident regional differences and provided variables that were more informative for origin discrimination than those in the higher-mass region. Prior GC-MS-based metabolomics reported volatile markers capable of distinguishing Xinhui CRP from other regions [38], and methyl N-methylanthranilate-related compounds have been recognized as aroma-associated constituents linked to authentic Xinhui CRP [39]. In the present work, DART-QTOF-MS captured methyl N-methylanthranilate without chromatographic separation, and its markedly higher relative abundance in Xinhui samples further supports its practical utility as a region-linked marker [39]. More broadly, the observation that LMW profiles were more informative than HMW profiles for geographic classification in the present dataset aligns with a growing body of food authentication literature, where VOCs and other small molecules often encode environmental and processing signatures and could support strong classification performance under chemometric or machine-learning modeling [40,41,42]. Overall, CRP shows a conserved core of genetically controlled major metabolites that underpins quality consistency, while LMW constituents might be more responsive to origin and processing conditions and therefore might offer greater discriminatory value in this context.

A central contribution of this study is the proposal of 2-indolinone as a potential novel marker for Xinhui CRP. Both DART-QTOF-MS fingerprints and targeted UPLC–MS/MS confirmation indicated that 2-indolinone was consistently enriched in multiple core Xinhui production zones, whereas signals in non-Xinhui samples were weak or barely detectable. This combination exemplifies a two-tier traceability workflow, in which DART-QTOF-MS enables high-throughput screening and orthogonal LC–MS/MS provides confirmatory verification for regulatory- or industry-facing decisions. To our knowledge, 2-indolinone has not previously been reported as a characteristic constituent of CRP. Importantly, its differential abundance between sample groups suggests that it captures origin-related information not reflected by the relatively stable PMF profile. Although VIP values are useful for evaluating variable contributions in the OPLS-DA model, they should not be regarded as the sole basis for marker selection. In the present study, marker prioritization also considered the consistency of inter-group differences, specificity for Xinhui CRP, feasibility of analytical confirmation, and literature support. From this perspective, 2-indolinone was considered particularly promising, whereas some compounds with relatively high VIP values may be less suitable as preferred markers because they are more widely distributed in CRP and are therefore less specific for authenticity assessment. Because 2-indolinone is an aromatic heterocycle potentially linked to indole metabolism, several plausible formation hypotheses can be advanced. Indole derivatives are ubiquitous in plants through tryptophan/auxin (IAA) metabolism, and citrus peel tissues have demonstrated oxidative IAA catabolism capacity, yielding oxindole-type derivatives such as oxindole-3-acetic acid and related products in Citrus sinensis peel [43]. In principle, downstream decarboxylation or further transformations of oxindole-type intermediates could contribute to free 2-indolinone formation, although direct evidence in CRP remains lacking. In parallel, microbial transformation routes may be relevant: 2-oxindole has been reported as a prominent metabolite produced by gut microbiota from tryptophan, implying that microbiome-associated catabolism of indole precursors can generate oxindole scaffolds under biologically realistic conditions [44]. Given that traditional CRP processing and storage may involve complex micro-environments, microbial contributions to LMW heterocycles deserve targeted evaluation.

Even if the exact formation pathway is unresolved, environmental and agro-ecological features characteristic of Xinhui provide a coherent context for why 2-indolinone might behave as a terroir-integrating marker. Soil conditions and plant–microbiome interactions have been linked to enhanced accumulation of monoterpenes in Citrus reticulata “Chachi” fruit in the core Xinhui production area [45]. Such findings support the broader idea that local environment and associated microbiota can shift the low-mass metabolome in ways that may be uncommon in non-Xinhui samples. Therefore, the observed enrichment trend of 2-indolinone in Xinhui CRP may reflect an integrated outcome of cultivar background, local environmental stressors, and processing/storage microbiology, rather than a single deterministic factor. These hypotheses are testable and motivate follow-up studies combining targeted metabolomics, microbiome profiling, and enzymology.

Chemometric evidence further suggests the central conclusion that LMW variables might provide more useful discriminatory information than HMW PMFs for subregional differentiation within Xinhui. In this study, OPLS-DA modeling using HMW flavonoid-dominated variables failed to separate core from non-core Xinhui regions, consistent with their broadly comparable levels across samples. In contrast, reconstruction of the OPLS-DA model using selected LMW compounds improved the separation trend relative to the higher-mass variables, indicating that LMW markers offer more robust information for finer-scale zoning. This observation aligns with recent reports that data-fusion strategies and machine-learning approaches could assist discrimination among multiple Xinhui production areas, supporting the feasibility of refined within-GI (geographical indication) classification frameworks [7].

Given that CRP is an aged-dried product, the effects of storage and ageing should be taken into consideration when interpreting geographic markers. In the present study, all samples were aged for 2 years, which reduced variation associated with vintage but limited evaluation of marker stability across different storage durations. Previous studies have shown that ageing can substantially remodel CRP chemical composition and bioactivity through compound transformation during storage [46,47,48,49,50]. Therefore, future work should determine whether key low-molecular-weight markers, including 2-indolinone and methyl N-methylanthranilate, remain stable or exhibit predictable changes under different ageing durations and storage conditions.

Several limitations of the current study should be acknowledged, together with priorities for method validation and standardization. First, although the sampling design reduced vintage effects by fixing ageing time, broader validation across harvest years, seasons, and ageing durations is required to establish marker generalizability and to quantify inter-annual variance. Second, DART-QTOF-MS primarily provides relative ion intensities, and compound-dependent ionization efficiencies can bias comparisons; therefore, targeted quantification by LC-MS/MS should be expanded to additional markers to improve transferability and support inter-laboratory reproducibility [51,52]. Third, the biochemical and/or microbiological origin of 2-indolinone remains unresolved. Because 2-indolinone was independently confirmed in the sample extracts by UPLC–MS/MS using an ESI source, its occurrence is unlikely to be attributable to artifact formation during the DART ionization process. Instead, it is more plausibly related to endogenous transformation within the CRP matrix or to reactions occurring during processing and/or storage, although dedicated mechanistic studies will be required to verify this. Finally, for routine authentication, standardizing key metadata and operating procedures, including sample pretreatment, moisture control, extract concentration, DART temperature, acquisition parameters, and chemometric pipeline, will be essential to ensure that models remain robust when deployed across laboratories and supply chains.

Despite these limitations, this work provides a concise and high-throughput workflow that integrates rapid DART-QTOF-MS fingerprinting with chemometric pattern recognition for origin-oriented characterization of CRP. Within this framework, we reconfirm the enrichment of methyl N-methylanthranilate in Xinhui CRP and propose 2-indolinone as a new candidate marker, providing a chemical basis for improving origin-discrimination models and informing refinement of quality standards for “Guang Chenpi” [53]. In the longer term, linking origin-related LMW markers with sensory attributes (aroma/flavor) and functional activities may further enhance their practical value for quality evaluation and value-added product development [54].

## 5. Conclusions

This study developed a rapid DART-QTOF-MS fingerprinting workflow combined with OPLS-DA for the geographical characterization of CRP. The results showed that high-mass ions, mainly represented by polymethoxylated flavones, constituted a relatively stable chemical background and contributed only modestly to regional discrimination. By contrast, low-molecular-weight ions carried more origin-related information, making them more effective for differentiating Xinhui CRP from samples of other origins and for probing subregional variation within Xinhui. Among these compounds, methyl N-methylanthranilate was reconfirmed as a Xinhui-enriched marker, while 2-indolinone was identified as a promising additional candidate marker and further verified by UPLC–MS/MS. Taken together, these results support the view that, for origin-oriented classification of CRP, the low-mass chemical region may offer greater practical value than the comparatively conserved high-mass region. Future studies should expand sampling across harvest years and storage conditions, strengthen targeted quantification, and clarify the formation mechanism and temporal stability of key low-molecular-weight markers to support broader validation and practical application.

## Figures and Tables

**Table 1:** The potential markers of Xinhui Citri Reticulatae Pericarpium.

	Compound	Molecular Formula	[M + H]^+^	VIP
M1	Methyl N-methylanthranilate	C_9_H_11_NO_2_	166.0856	2.0459
M2	2-Indolinone	C_8_H_7_NO	134.0593	1.3305
M5	2-Acetylpyrrole	C_6_H_7_NO	110.0576	1.2646
M6	Levoglucosenone	C_7_H_11_O_2_	128.1051	1.2420
M4	Octanal	C_8_H_16_O	130.0865	0.9767
M11	5,7,4′-Trihydroxy-8-methyldihydroflavone	C_16_H_14_O_5_	288.0820	0.8830
M12	Unknown	-	313.0651	0.7773
M9	Unknown	-	234.1073	0.7724
M10	Dihydroferulic acid	C_20_H_22_O_8_	197.1262	0.7454
M14	Tangeretin	C_20_H_20_O_7_	373.1243	0.7329
M13	3′,4′,5,7-Tetramethoxyflavone	C_19_H_18_O_6_	343.1127	0.5797
M8	4-Phenylbutyric acid	C_10_H_11_O_2_	164.1053	0.5616
M3	5,6-Dihydroxyindole	C_8_H_7_NO_2_	150.0888	0.3941
M7	5-Isopropyl-2-methyl pyrazine	C_8_H_12_N_2_	137.1054	0.2594

## Data Availability

The original contributions presented in this study are included in the article/Supplementary Materials. Further inquiries can be directed to the corresponding author.

## Supplementary Materials

The following supporting information can be downloaded at https://www.mdpi.com/article/10.3390/foods15081361/s1. Figure S1. Total ion current (TIC) profile of a methanolic extract of Citri Reticulatae Pericarpium acquired by DART-Q-TOF-MS; Figure S2. Mass spectra of potential marker ions (*m*/*z* 50–650); Figure S3. Mass spectra of potential marker ions (*m*/*z* 50–320, enlarged view); Figure S4. Mass spectra of potential marker ions (*m*/*z* 440–650); Figure S5. OPLS-DA score plot for high-molecular-weight compounds (R, samples from the core production region; S, samples from non-core production regions); Figure S6. Permutation test of the OPLS-DA model for high-molecular-weight compounds; Table S1. Material information of Xinhui CRP; Table S2. Concentrations (μg/kg) of 2-indolinone in Citri Reticulatae Pericarpium samples from different regions (ND = not detected); Table S3. High-molecular-weight compounds with low VIP (values).
