# Supplementary material for: Geographical Discrimination of Xinhui Citri Reticulatae Pericarpium by DART-QTOF-MS
Source: Foods. 2026 Apr 14;15(8):1361. doi: 10.3390/foods15081361 (PMC13115399; doi:10.3390/foods15081361)
Supplement: Supplementary file 1 [file foods-15-01361-s001.zip › foods-4179705-supplementary.pdf]

Geographical Discrimination of Xinhui Citri Reticulatae Pericarpium by DART-QTOF-MS

Ximei Wu [1,*,†], Qunjie Feng [1,†], Wenbo Duan [2], Wei Tong [1], Jian Wen [1] and Gangqiang Ding [1]

[1] NHC Specialty Laboratory of Food Safety Risk Assessment and Standard Development, Guangdong Provincial Center for Disease Control and Prevention, Guangzhou 511430, China
[2] Carbon Neutrality and Climate Change Thrust, Society Hub, The Hong Kong University of Science and Technology (Guangzhou), Guangzhou 511453, China
* Correspondence: wxmdwb927@163.com
† These authors contributed equally to this work.

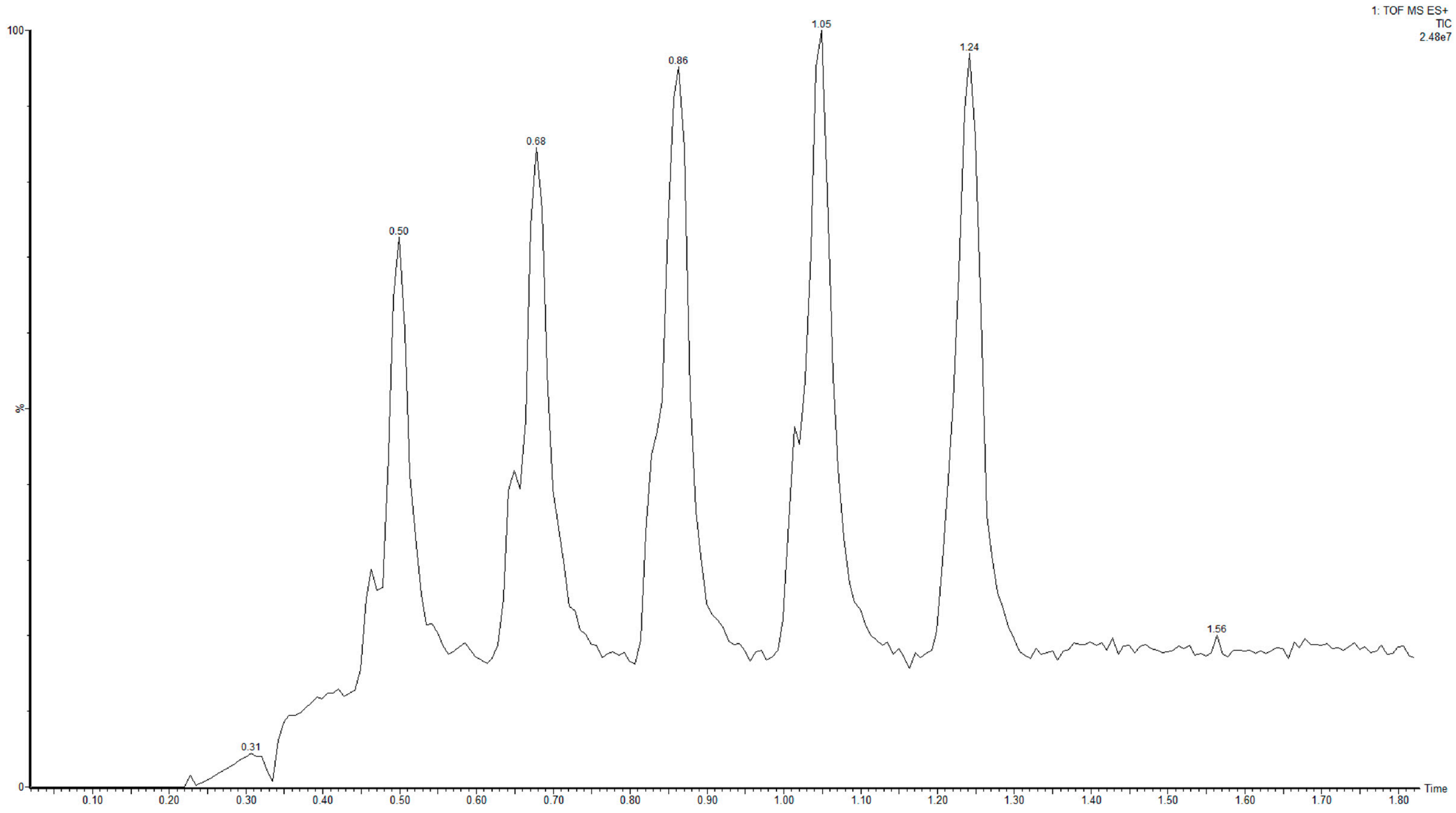

Figure S1. Total ion current (TIC) profile of a methanolic extract of Citri Reticulatae Pericarpium acquired by DART-Q-TOF-MS.

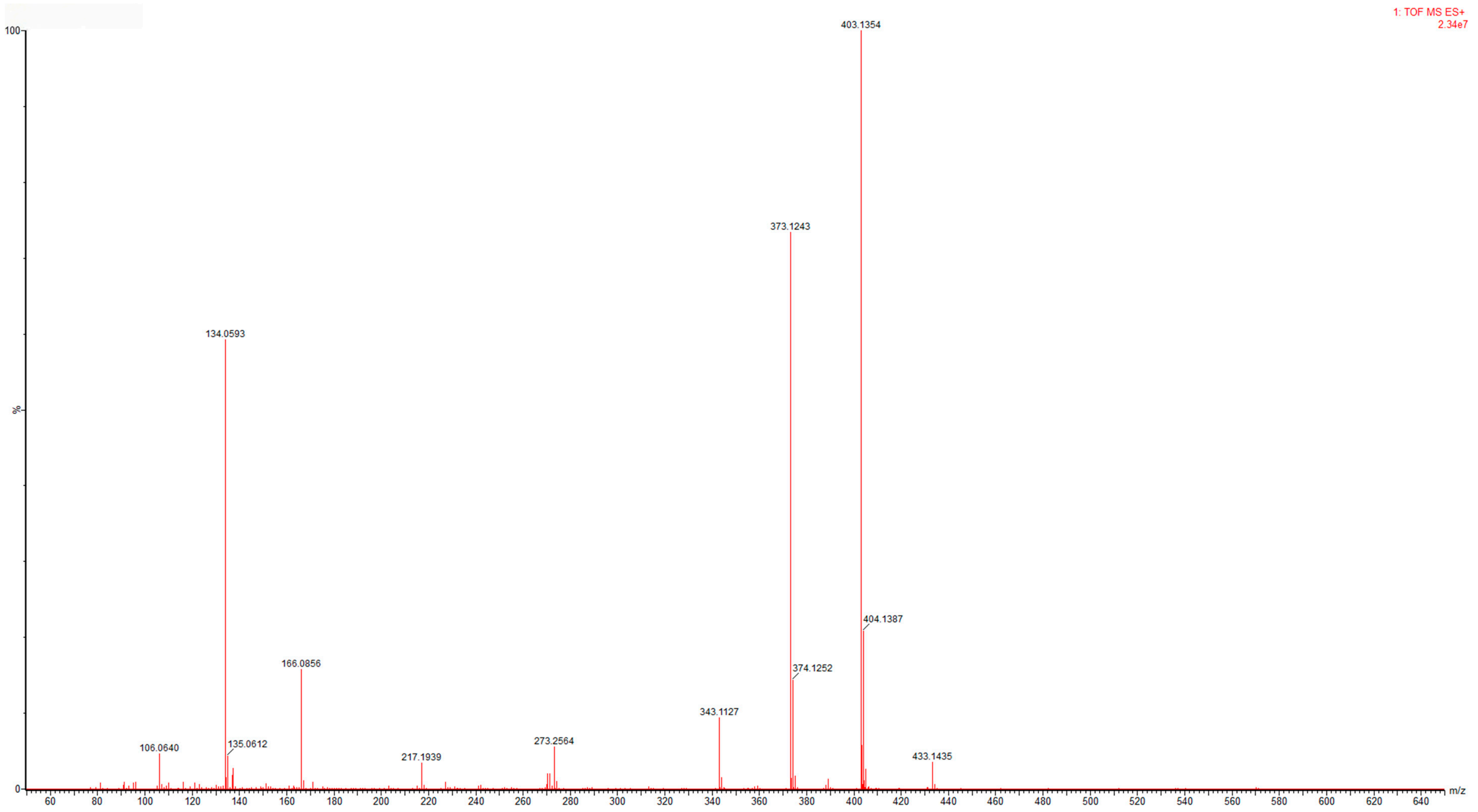

Figure S2. Mass spectra of potential marker ions (m/z 50–650).

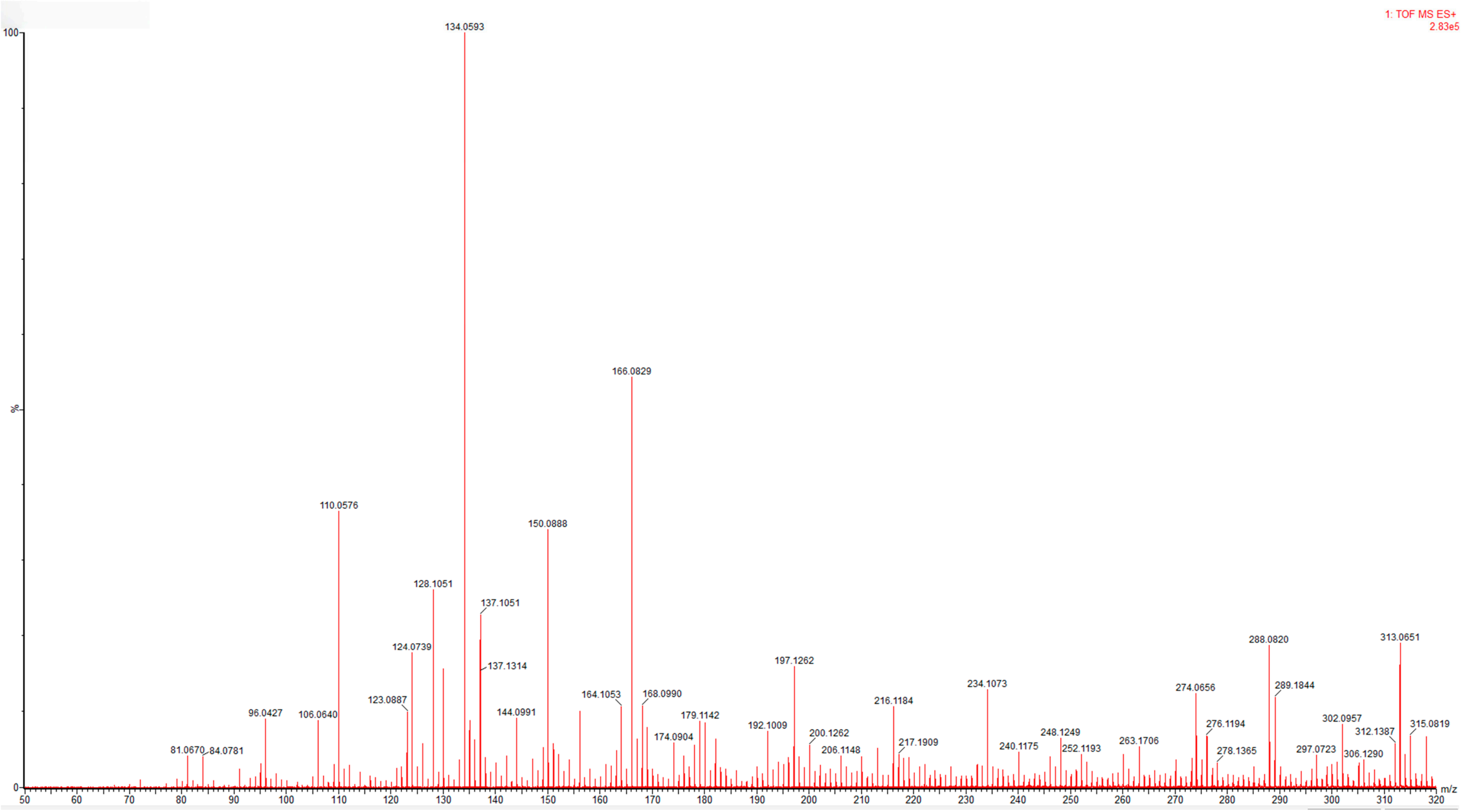

Figure S3. Mass spectra of potential marker ions (m/z 50–320, enlarged view).

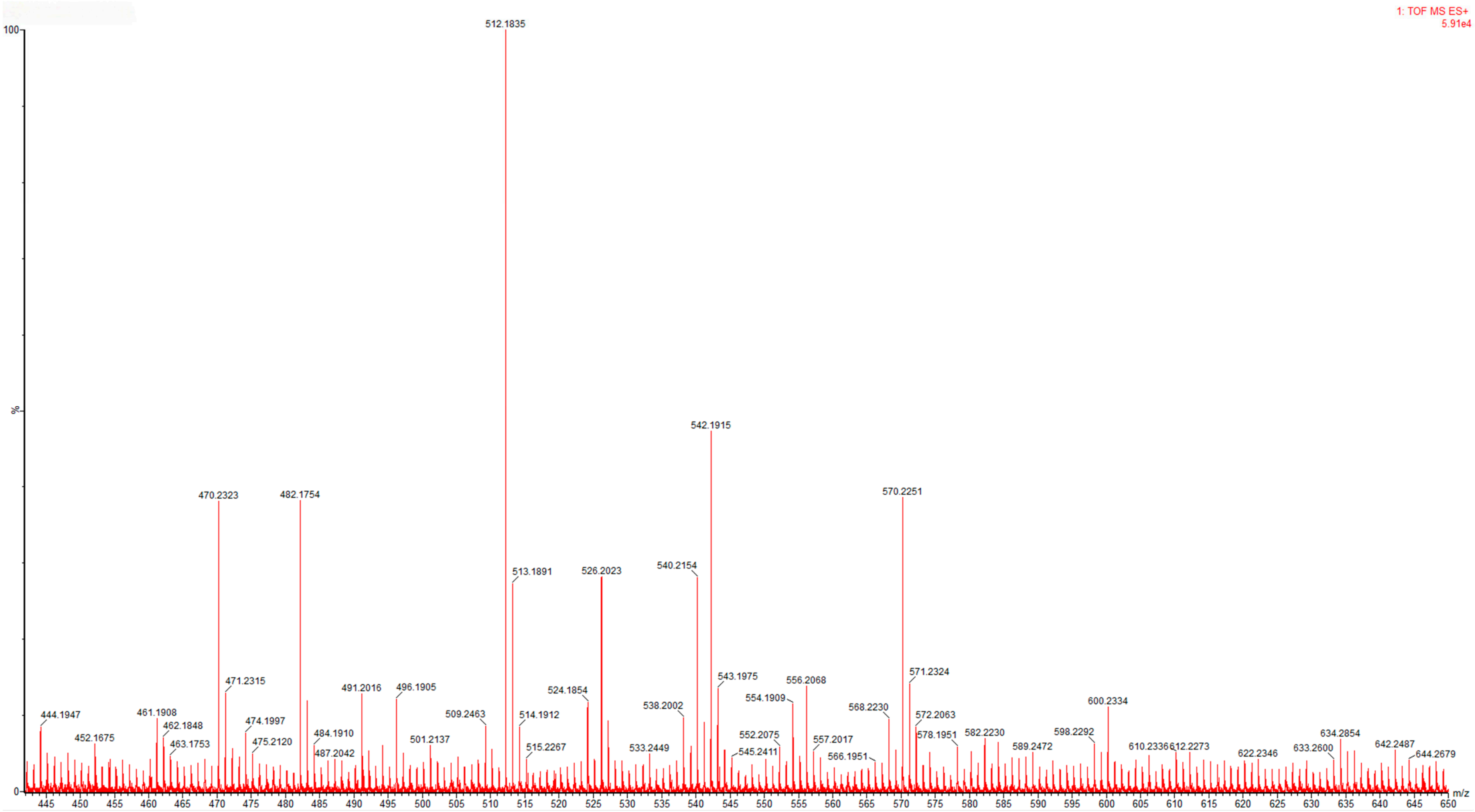

Figure S4. Mass spectra of potential marker ions (m/z 440–650).

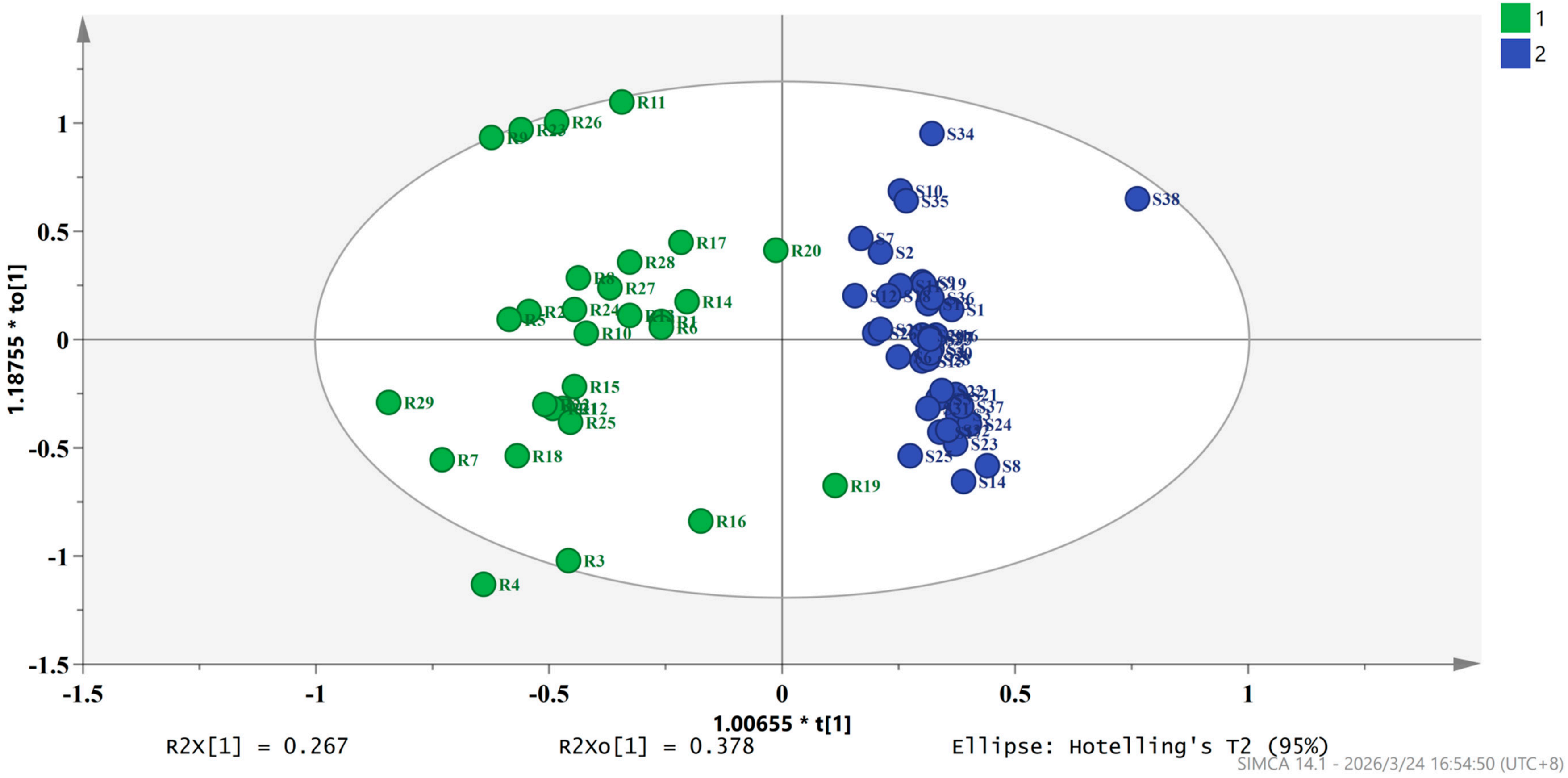

Figure S5. OPLS-DA score plot for high-molecular-weight compounds (R, samples from the core production region; S, samples from non-core production regions).

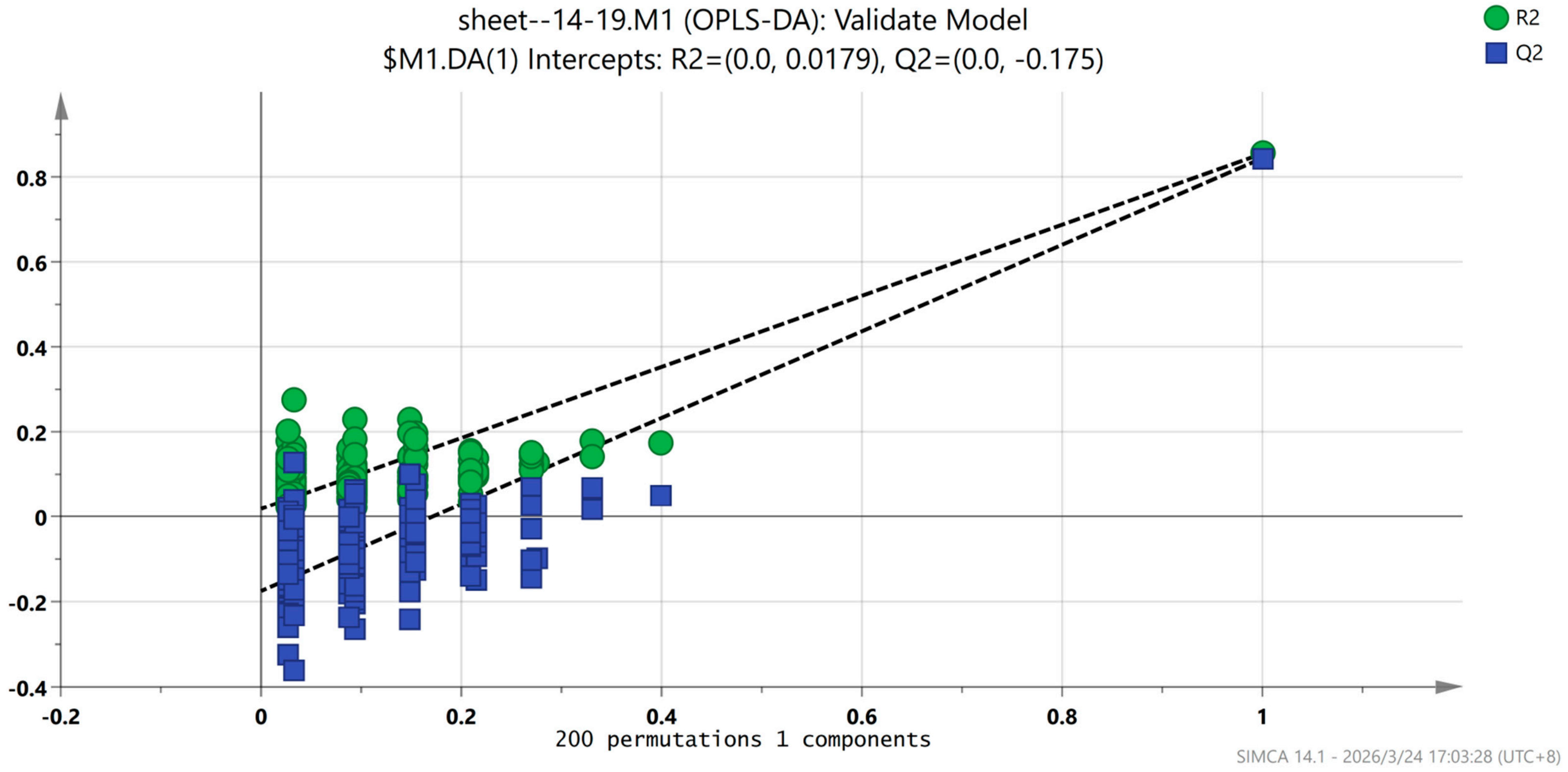

Figure S6. Permutation test of the OPLS-DA model for high-molecular-weight compounds

Table S1. Materialinformation of Xinhui CRP.

Samples	Region	Number	Harvest Year	Storage years
core production regions	Xinhui TianmaTown, Guangdong	8	2021	2
core production regions	Xinhui MeijiangTown, Guangdong	6	2021	2
core production regions	Xinhui Dongjia Town, Guangdong	7	2021	2
core production regions	Xinhui Chakeng Town, Guangdong	8	2021	2
non-core production regions	Xinhui SiqianTown, Guangdong	12	2021	2
non-core production regions	Xinhui YamenTown, Guangdong	10	2021	2
non-core production regions	Xinhui GujingTown, Guangdong	16	2021	2

Table S2. Concentrations (μg/kg) of 2-indolinone in Citri Reticulatae Pericarpium samples from different regions (ND = not detected).

Samples	Region	2-Indolinone
CRP xinhui	Xinhui District, Guangdong	99-476
CRP-1	Jinhua City,Zhejiang Province	30.5
CRP-2	Wuhu City Anhui Province	ND
CRP-3	Chenzhou City,Hunan Province	ND
CRP-4	Guangxi , Wuzhou City,Guangxi Province	16.3
CRP-5	Huizhou City, Guangdong Province	22.9
CRP-6	Heyuan City, Guangdong Province	36

Table S3. High-molecular-weight compounds with low VIP (values)

Compound	Molecular Formula	$[M+H]^+$
M12	Unkown	313.0551
M13	$C_{19}H_{18}O_6$	343.1127
M14	$C_{20}H_{20}O_7$	373.1243
M15	$C_{27}H_{29}NO_9$	512.1835
M16	$C_{24}H_{31}NO_{13}$	542.1915
M17	$C_{26}H_{31}NO_7$	470.2323
M18	$C_{26}H_{27}NO_8$	482.1754

M19	$C_{40}H_{56}O_2$	570.2251
